# Seasonality and Day-to-Day Variability of Dietary Diversity: Longitudinal Study of Pregnant Women Enrolled in a Randomized Controlled Efficacy Trial in Rural Burkina Faso

**DOI:** 10.1093/jn/nxac104

**Published:** 2022-05-07

**Authors:** Giles T Hanley-Cook, Alemayehu Argaw, Brenda de Kok, Laeticia Celine Toe, Trenton Dailey-Chwalibóg, Moctar Ouédraogo, Patrick Kolsteren, Lieven Huybregts, Carl Lachat

**Affiliations:** Department of Food Technology, Safety and Health, Faculty of Bioscience Engineering, Ghent University, Ghent, Belgium; Department of Food Technology, Safety and Health, Faculty of Bioscience Engineering, Ghent University, Ghent, Belgium; Department of Food Technology, Safety and Health, Faculty of Bioscience Engineering, Ghent University, Ghent, Belgium; Department of Food Technology, Safety and Health, Faculty of Bioscience Engineering, Ghent University, Ghent, Belgium; Institut de Recherche en Sciences de la Santé (IRSS), Unité Nutrition et Maladies Métaboliques, Bobo-Dioulasso, Burkina Faso; Department of Food Technology, Safety and Health, Faculty of Bioscience Engineering, Ghent University, Ghent, Belgium; AFRICSanté, Bobo-Dioulasso, Burkina Faso; Department of Food Technology, Safety and Health, Faculty of Bioscience Engineering, Ghent University, Ghent, Belgium; Department of Food Technology, Safety and Health, Faculty of Bioscience Engineering, Ghent University, Ghent, Belgium; Poverty, Health and Nutrition Division, International Food Policy Research Institute (IFPRI), Washington, DC, USA; Department of Food Technology, Safety and Health, Faculty of Bioscience Engineering, Ghent University, Ghent, Belgium

**Keywords:** balanced energy-protein supplements, Burkina Faso, dietary diversity, food groups, list-based recall, pregnant women, seasonality

## Abstract

**Background:**

Panel data indicate that nonpregnant women's dietary diversity fluctuates across climatic seasons in low- and middle-income countries. The natural day-to-day variability in food group consumption during gestation is unknown.

**Objectives:**

A longitudinal study was conducted among pregnant women enrolled in the Micronutriments pour la Santé de la Mère et de l'Enfant study 3 randomized controlled efficacy trial [i.e., daily fortified balanced energy-protein supplement and an iron-folic acid (IFA) tablet compared with an IFA tablet only] to investigate the number of 24-hour recalls required to estimate usual prenatal food group (FG) diversity and the seasonality of pregnant women's dietary diversity in Houndé, Burkina Faso.

**Methods:**

FG consumption was assessed twice weekly by qualitative, list-based, 24-hour recalls among 1757 pregnant women (892 control, 865 intervention). The number of days needed to estimate a woman's usual prenatal 10-point FG diversity score was calculated using the within-subject coefficient of variation. Regression models, including truncated Fourier series, were fitted to assess seasonal variations in the FG diversity score and the probability of reaching Minimum Dietary Diversity for Women (MDD-W; i.e., ≥5 FGs).

**Results:**

The monthly mean FG scores (<5 FGs) and MDD-W prevalence (<45%) were low. Five list-based recalls allowed observed FG diversity to lie within 15% of the true mean in 90% of the estimations (mean ± SD, 40.4 ± 20.7 recalls per woman). Both the FG diversity score and prevalence achieving MDD-W showed responsiveness to seasonal variations, with peaks at the end of the dry season (i.e., April or May) and troughs in the rainy season (i.e., August).

**Conclusions:**

Five list-based recalls are sufficient to estimate usual FG diversity during gestation, although intra-annual seasonal patterns did modestly affect the FG diversity score and MDD-W prevalence. Thus, timing of repeated dietary surveys is critical to ensure nonbiased inferences of change and trends in Burkina Faso. This trial was registered at clinicaltrials.gov as NCT 03533712.

## Introduction

During pregnancy, women have increased requirements for energy and nutrients to support changes in maternal physiology (i.e., metabolism and tissue growth) and fetal development (1). In many populations in low- and middle-income countries (LMICs), diets are habitually nondiverse and dominated by micronutrient-poor, starchy staples, and consequently fail to provide adequate amounts of (bioavailable) micronutrients (2–4). Nondiverse prenatal diets lead to nutrient deficiencies and, subsequently, adverse health outcomes in both the mother and newborn (5). Antenatal care guidance from the WHO upholds the critical role of dietary diversity during gestation (6). Nevertheless, in settings with a high prevalence of women who are underweight (BMI <18.5 kg/m²), providing pregnant women with fortified balanced energy-protein (BEP) supplements (i.e., <25% of total kcal from protein) is advised to fill nutrient gaps and to reduce the risk of stillbirths and small-for-gestational-age neonates (7).

Food group (FG) diversity scores, which consist of counting the number of FGs recalled over a defined period (e.g., 24 hours), are relatively straightforward to measure and are widely used as population-based indicators to assess dietary diversity in multitopic surveys (e.g., Demographic and Health Surveys) (8) and intervention studies (9). In 2016, the 5 out of 10 FG cutoff of the Minimum Dietary Diversity for Women (MDD-W) indicator was identified as the most accurate cutoff to predict a mean probability of adequacy (MPA) >0.60 of nonpregnant and lactating women's diets across 11 micronutrients (10). Moreover, studies on prenatal FG diversity scores and, more recently, the dichotomous MDD-W indicator have resulted in prospective, inverse associations with adverse birth outcomes (e.g., preterm birth, low birth weight) in LMICs (11–15). However, single-day recalls ignore random within-person variability and are thus not representative of a woman's usual FG consumption or MDD-W (16). Therefore, when single-time-point FG diversity scores are used in regression analyses to study associations with health outcomes, they might lead to attenuated model coefficients (17). To our knowledge, no study has assessed the minimum number of repeated recalls required to precisely estimate an individual's usual FG diversity score.

Seasonal patterns often affect food availability, affordability, and accessibility in rural populations (18), which poses inherent challenges to single-time-point assessments of dietary diversity (i.e., undetermined within-subject variance). At present, most studies investigating women's dietary diversity rely on cross-sectional surveys, with or without intra-annual data collection, which likely lead to inaccurate and imprecise estimates of usual FG diversity (19). Furthermore, in Africa, seasonal variations in adverse birth outcomes (e.g., lower birth length) have also been attributed to fluctuations in prenatal maternal nutritional status, likely due to periodical food shortages and higher energy expenditure related to agricultural labor (20,21) that coincide with seasonal epidemics of infectious and parasitic diseases (22). Therefore, the consideration of seasonal fluctuations in dietary diversity might allow for more accurate causal analyses of nutrition interventions and for better monitoring and evaluation of food and nutrition policy impacts, and could, as such, contribute to better targeting and timing of dietary interventions.

Our research team conducted a randomized controlled efficacy trial [Micronutriments pour la Santé de la Mère et de l'Enfant study 3 (MISAME-III)] in which a daily, fortified BEP and iron-folic acid (IFA) tablet was compared to an IFA tablet only in rural Burkina Faso, with the primary outcome of reducing the small-for-gestational age prevalence. Concurrently, qualitative, list-based, 24-hour recalls were enumerated multiple times per week. Hence, high-frequency FG consumption data were collected among enrolled pregnant women to systematically gauge their diets (23). This longitudinal study's objective was bipartite. First, we sought to identify the number of qualitative, list-based recalls required to estimate the usual prenatal FG diversity score. Second, we sought to assess seasonal effects on pregnant women's dietary diversity in Houndé, Burkina Faso.

## Methods

Our research was reported using the Strengthening the Reporting of Observational Studies in Epidemiology–Nutritional Epidemiology checklist (24).

### Data source

We used data from the MISAME-III randomized controlled efficacy trial (23). In brief, 1788 pregnant women were individually, randomly assigned to the prenatal intervention group or control group in permuted blocks of 8. Randomization codes were sealed in opaque envelopes by a person not participating in the implementation of the trial. After written informed consent was obtained from eligible participants, study midwives opened the next envelope, assigned the pregnant woman to 1 of 2 prenatal trial arms, and transmitted the assignment codes and personal identifiers to the person responsible for the daily supplement distribution. The intervention group received a daily fortified BEP supplement and an IFA tablet, and the control group received an IFA tablet alone. Daily BEP and IFA intake was directly observed by the trained, village-based project workers. In a formative study, the most preferred and suitable fortified BEP was selected for administration in the randomized controlled trial (25,26). Anthropometric and sociodemographic data were collected at baseline. The MISAME-III trial enrolment ran between 30 October 2019 and 12 December 2020.

### Study population and area

Prenatal FG intake data were collected from 30 October 2019 through 6 August 2021 in 6 rural health-center catchment areas located in the Houndé health district in the Hauts-Bassins region of Burkina Faso. The climate of the region is Sudano-Sahelian. Conventionally, the rainy season runs from May to September or October and is characterized by a seasonal increase in agricultural labor and energy expenditure, as well as food scarcity because of diminishing food stocks. The arrival of the first rains in early June is the sign for many rural households to start sowing, whereas the period around October is dedicated to harvesting and marks the beginning of the dry season, which runs through to April. In 2020, single-day, multiple-pass, quantitative, 24-hour recalls conducted by our research team among 470 pregnant women (253 control, 217 intervention) estimated the mean energy intake of the base diet (i.e., excluding supplements) to be ∼1940 kcal at the end of the preharvest season (27). Moreover, rural Burkinabe diets are nondiverse and cereal-based (28) and, hence, frequently fail to cover the estimated average requirements of daily micronutrient intakes during pregnancy (27,29). Maize is the main staple food and is harvested in October and November. The main economic activity is agriculture, which is focused on family farming and rain-fed crops, especially cotton and maize.

### Dietary assessment

Trained, village-based project workers assessed pregnant women's FG consumption in the local language at least twice weekly over the prenatal follow-up, on a random day, by means of a nonquantitative, list-based, 24-hour recall conducted using a mobile phone–based strategy implemented in the Census and Survey Processing System (CSPro) software (version 7.3.0; US Census Bureau).

To ensure that only foods usually consumed in daily quantities at or above the 15-g threshold were included (i.e., ∼1 tablespoon), the food list (English translation in **Supplemental Table 1**) was developed based on consultations with (local) nutritionists and health workers and on data from an earlier quantitative dietary intake study by Huybregts et al. (29) among nonpregnant and pregnant women in Houndé.

Unfortunately, due to a programming error of our CSPro date variable, a woman's FG consumption data over the prenatal follow-up were aggregated by the month of recall (mm/yyyy format). To clarify, our panel study initially aimed to collect daily FG consumption data (dd/mm/yyyy format); however, multiple list-based recalls collected within the same month (e.g., on 7 May 2021 and 14 May 2021) were both exported with a nonunique date identifier (e.g., May 2021). Consequently, an average FG diversity score was computed from the list-based recalls collected on different days (i.e., unique metadata, such as the interview number) within a single month (e.g., 4, 7, 6, 4, 3, and 5 out of 10 FGs enumerated in May 2021 were collapsed to a mean of 4.8 FGs).

### FG diversity score and the MDD-W indicator

For the list-based recall, the 11 FGs enumerated (i.e., vitamin A–rich fruits and vegetables were recalled separately; Supplemental Table 1) were aggregated into the 10 predefined MDD-W FGs (30): *1*) starchy staple foods; *2*) beans and peas; *3*) nuts and seeds; *4*) dairy products (milk, yogurt, and cheese); *5*) flesh foods (meat, fish, poultry, and liver or organ meats); *6*) eggs; *7*) dark green, leafy vegetables; *8*) vitamin A–rich fruits and vegetables; *9*) other vegetables; and *10*) other fruits. The FG diversity score (0–10) was constructed by summing the number of FGs consumed at a specific recall. Reaching the MDD-W was defined as a pregnant woman consuming ≥5 FGs. Moreover, following evidence by Nguyen et al. (31) of alternative cutoffs (i.e., MPA >0.60) for dietary micronutrient adequacy during pregnancy, we also assessed the proportion of pregnant women consuming ≥6 FGs in the previous 24 hours.

### Statistical analysis

Data management and the statistical analysis were performed in Stata (version 16.1; StataCorp). Following the eligibility criteria of MISAME-III, only women with singleton pregnancies enrolled at ≤20 weeks of gestation and from whom at least 1 list-based, 24-hour recall was enumerated were included in the analyses.

Baseline characteristics of participants, by study arm, and pooled monthly dietary diversity variables were summarized as means ± SDs or medians (IQRs) for continuous variables and as frequencies (percentages) for categorical variables. Of note is that median values of the pseudocontinuous (i.e., an ordinal variable statistically assessed as if continuous) FG diversity score are presented to the first decimal place (e.g., 3.6 FGs) due to aggregation of list-based, 24-hour recall data by study month (see above). We compared the average monthly 10-point FG diversity scores and the prevalence reaching MDD-W between the control and intervention groups using Welch's independent-sample *t*-test. In addition, between- and within-person SDs of the 10-point FG diversity score and the average number of observations per woman over the efficacy trial's prenatal follow-up were calculated using Stata's *loneway* command. Moreover, to determine the number of qualitative, list-based recalls needed for the observed FG diversity to lie within 15% of the true (nonaggregated) mean in 90% of the estimations, we used the following formula (32):

(1) \begin{eqnarray*} {\rm{d}} = {({{\rm{Z}}_\alpha } \times {\rm{C}}{{\rm{V}}_{{\rm{intra}}}}/{\rm{D}})^2} \end{eqnarray*}

Here, d is the number of replications required; Z*_α_* is the z-statistic (i.e., 1.645); CV*_intra_* is the within-subject CV as a percentage, calculated as the within-subject SD (from women with at least 2 list-based, 24-hour recalls only) divided by the mean FG diversity; and D is the specified error, given as a percentage of the true usual intake. We repeated the estimation among pregnant women with recall data from dry- or rainy-season months only and from those with recall data across both seasons.

Prior analyses of the seasonality of individual- or household-level dietary diversity range from (fixed- or mixed-effects) regression (19,33–39) to time-series summaries of (bi)monthly (19,40,41) or seasonal values (34, 39, 42–52), followed by the fit of a regression model that takes a 12-month periodicity (annual model) into account (8,40), and models including an interaction term between dietary diversity indices and survey timing to assess effect modification on nutrient intake (47,54,55). However, those methods can lead to over-parameterization or may introduce abrupt changes by the arbitrary choice of seasonal cutoffs (e.g., lean compared with plenty). Following previous statistical modeling studies of the seasonality of birth outcomes (20,56), we modeled the seasonal trends of the pooled (i.e., both control and intervention arms) monthly FG diversity score and MDD-W with truncated Fourier terms (i.e., a periodic function of sinusoids).

**TABLE 1:** Baseline characteristics of pregnant women, by MISAME-III trial arm^1^

Characteristics	Control (*n* = 892)	Intervention (*n* = 865)
Health center catchment area		
Boni	196 (22.0)	188 (21.7)
Dohoun	94 (10.5)	97 (11.2)
Dougoumato II	169 (19.0)	153 (17.7)
Karaba	92 (10.3)	93 (10.8)
Kari	160 (17.9)	158 (18.3)
Koumbia	181 (20.3)	176 (20.4)
Household		
Wealth index, 0–10 points	4.52 ± 1.74	4.67 ± 1.76
Farming household	640 (71.7)	638 (73.8)
Household food insecurity^2^	481 (53.9)	482 (55.7)
Improved primary water source^3^	554 (62.1)	542 (62.7)
Improved sanitation facility^4^	531 (59.5)	530 (61.3)
Insecticide-impregnated bed net	721 (80.8)	674 (77.9)
Household size	6.20 ± 4.45	6.20 ± 4.22
Monogamous	399 (44.7)	387 (44.7)
Polygamous	286 (32.1)	285 (32.9)
Head of household		
Age, years	33.5 ± 9.15	33.8 ± 9.34
Male	99.7	99.8
Completed primary education	59.9	58.8
Women		
Age, years	25.2 ± 6.21	24.9 ± 6.18
Ethnic group		
Bwaba	508 (57.0)	494 (57.1)
Mossi	318 (35.7)	302 (34.9)
Other	66 (7.39)	69 (7.98)
Religion		
Muslim	378 (42.4)	370 (42.8)
Animist	205 (23.1)	199 (23.1)
Protestant	145 (16.3)	154 (17.8)
Catholic	129 (14.5)	112 (13.0)
No religion, no animist	34 (3.81)	28 (3.24)
Completed primary education	376 (42.1)	360 (41.6)
Weight, kg	58.0 ± 8.65	58.3 ± 8.65
Height, cm	162 ± 5.91^5^	163 ± 6.05
BMI, kg/m²	22.0 ± 2.88	22.0 ± 2.85
<18.5 kg/m²	60 (6.73)	63 (7.28)
Midupper arm circumference, mm	263 ± 26.9	262 ± 26.2
Subscapular skinfold, mm	11.9 ± 5.50	12.0 ± 5.57
Tricipital skinfold, mm	11.8 ± 4.78	12.0 ± 4.84
Hb, g/dl	11.4 ± 1.47	11.3 ± 1.52
Anemia (Hb <11g/dl)	327 (36.7)	334 (38.6)
Severe anemia (Hb <7g/dl)	2 (0.22)	2 (0.23)
Gestational age, weeks	11.4 ± 4.05	11.5 ± 4.03
Trimester of gestation		
First	567 (63.6)	538 (62.2)
Second	325 (36.4)	327 (37.8)
Parity		
0	191 (21.4)	200 (23.1)
1–2	318 (35.7)	291 (33.6)
≥3	383 (42.9)	374 (43.2)

1 Data are *n* (%) or mean ± SD. Abbreviations: Hb, hemoglobin; MISAME-III, Micronutriments pour la Santé de la Mère et de l'Enfant study 3.

2 Assessed using FANTA/USAID's Household Food Insecurity Access Scale (53).

3 Protected-well, borehole, pipe, or bottled water were considered improved water sources.

4 A flush toilet connected to local sewage or septic tank or a pit latrine with slab and/or ventilation were considered improved sanitation facilities.

5 The height of 1 woman with a physical disability could not be measured.

Dates (mm/yyyy format) of list-based, 24-hour recalls were transformed into cyclic data (i.e., a continuous variable with circular distribution), with the starting point set as January. Each date was represented by an angle *θ_i_* = 2π (*D_i_* mod 12)/12, expressed in radians, so that the 2π radians cover a year (i.e., 12 months). *D_1_* is the number of months between January 1960 and the *i*^th^ pregnant woman's dietary intake assessment. The first *p* pairs of term of the Fourier series are include in the regression models as follows:

(2) \begin{eqnarray*} S\left( {{\theta _i},\ p} \right)\ = \ \sum\limits_{r = 1}^p {{\beta _r}\sin \left( {r{\theta _i}} \right)\ + \ {\gamma _r}\cos \left( {r{\theta _i}} \right)} \end{eqnarray*}

Here, *r* is the order of the Fourier term. Seasonal effects acting at the time of FG consumption are modeled by adding *S*(*θ_i_, p*) to the linear predictor so that *β_i_* and *γ_i_* become parameters in a regular, multivariable regression model. Pairs of Fourier terms (sine and cosine of the same order) were included in a regression model by increasing order, starting with the first-order pair up to the third-order pair. The first-order terms (sin*θ* and cos*θ*) model 6-month cycles, the second-order terms (sin2*θ* and cos2*θ*) model 3-month cycles, and the third-order terms (sin3*θ* and cos3*θ*) model 1.5-month cycles. Fourier terms (sine and cosine) of the same order represent the same period and are orthogonal. Therefore, if 1 of these components is significantly associated with the outcome of interest, the order is also considered to be significantly relevant to this outcome. Regression coefficients are interpreted as in a simple regression equation, except for the coefficients of Fourier terms that must be interpreted conjointly. The interpretation of these models can be best explained using an example. Assume that after convergence, the model for the 10-point FG diversity score over time of list-based, 24-hour recalls would be ŷ = 4.5 + 0.1sin*θ* – 0.2cos*θ* + e. We calculate that in July, or at *θ* = π radians, the predicted average FG diversity score would be 4.5 + 0.1 × 1 + (−0.2) × 0 = 4.6, whereas in April, or at π/2 radians, 4.5 + 0.1 × 0 + (−0.2) × 1 results in a FG diversity score of 4.3.

To determine which orders of Fourier terms fully captured the seasonality of the FG diversity score, higher-order models were compared to lower-order models using the likelihood-ratio chi-square test (*P* < 0.05) and, in addition, the Akaike information criterion (AIC) and Bayesian information criterion (BIC). Models that include up to first-, second-, or third-order Fourier terms are respectively named F_1_, F_2_, or F_3_ models.

Seasonal trends in the pseudocontinuous outcome FG diversity score (0−10) and monthly aggregated binary outcome MDD-W (i.e., range 0−1) were analyzed by fitting linear mixed-effects regression models (random intercept: woman). The models only included Fourier terms as predictors to model the crude trend of seasonality in pregnant women's FG diversity scores.

All statistical tests were 2-sided, and significance was set at a *P* value < 0.05, except for interactions for which a *P* value < 0.10 was used.

### Ethical considerations

The MISAME-III trial was approved by the Ethics Committee of Ghent University Hospital (B670201734334) and the Burkinabé Ethics Committee of Centre Muraz (no. 2018–22/MS/SG/CM/CEI). Written informed consent was obtained from all participants before enrolment.

## Results

A total of 1788 pregnant women were randomly allocated to the 2 prenatal study arms: 909 to the control group (daily IFA) and 879 to the intervention group (daily BEP and IFA). Five women (4 control, 1 intervention) never received a home visit due to relocation to another health-center catchment area; whereas 10 women in the control group and 16 in the intervention group did not complete a single prenatal qualitative list-based, 24-hour recall. Hence, our longitudinal study was conducted among 1757 pregnant women (892 control, 865 intervention; **Supplemental Figure 1**).

Women's characteristics were similar [i.e., <2.5 percentage point (pp) difference in absolute values] between the prenatal study arms at enrolment (**Table 1**). In summary, ∼40% of women completed at least primary education and >70% of households identified as (subsistence) agriculturalists. Overall, >50% of households were food insecure and >35% of pregnant women were anemic at baseline (hemoglobin <11 g/dL). At least 2 list-based, 24-hour recalls were collected from 1743 pregnant women (99.2%), whereas 1468 (83.6%) provided FG consumption data from months in both the rainy and dry seasons. On average, prenatal FG intake was followed up over 6.2 months.

**FIGURE 1:**
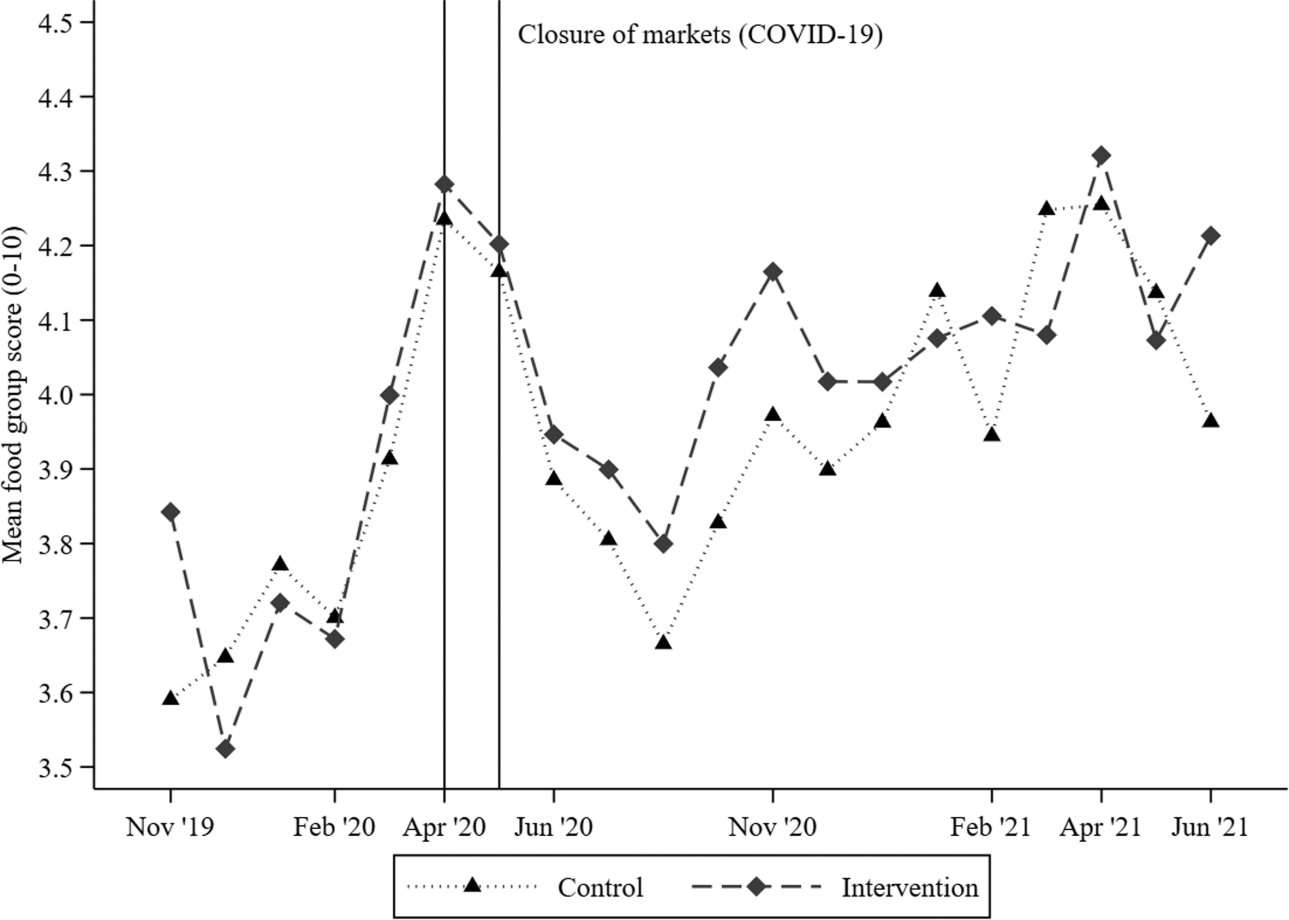
Food group diversity score among pregnant women (*n* = 1757), by study month and trial arm. Y-axis ranges between a minimum of 3.5 and a maximum of 4.5 food groups. October 2019 (*n* = 8 women), July 2021 (*n* = 14), and August 2021 (*n* = 1) were not plotted due to the limited number of data points. All 2-sided Welch's independent-sample *t*-tests were nonsignificant by intervention arm (*P* > 0.05). Abbreviation: COVID-19, coronavirus disease 2019.

**FIGURE 2:**
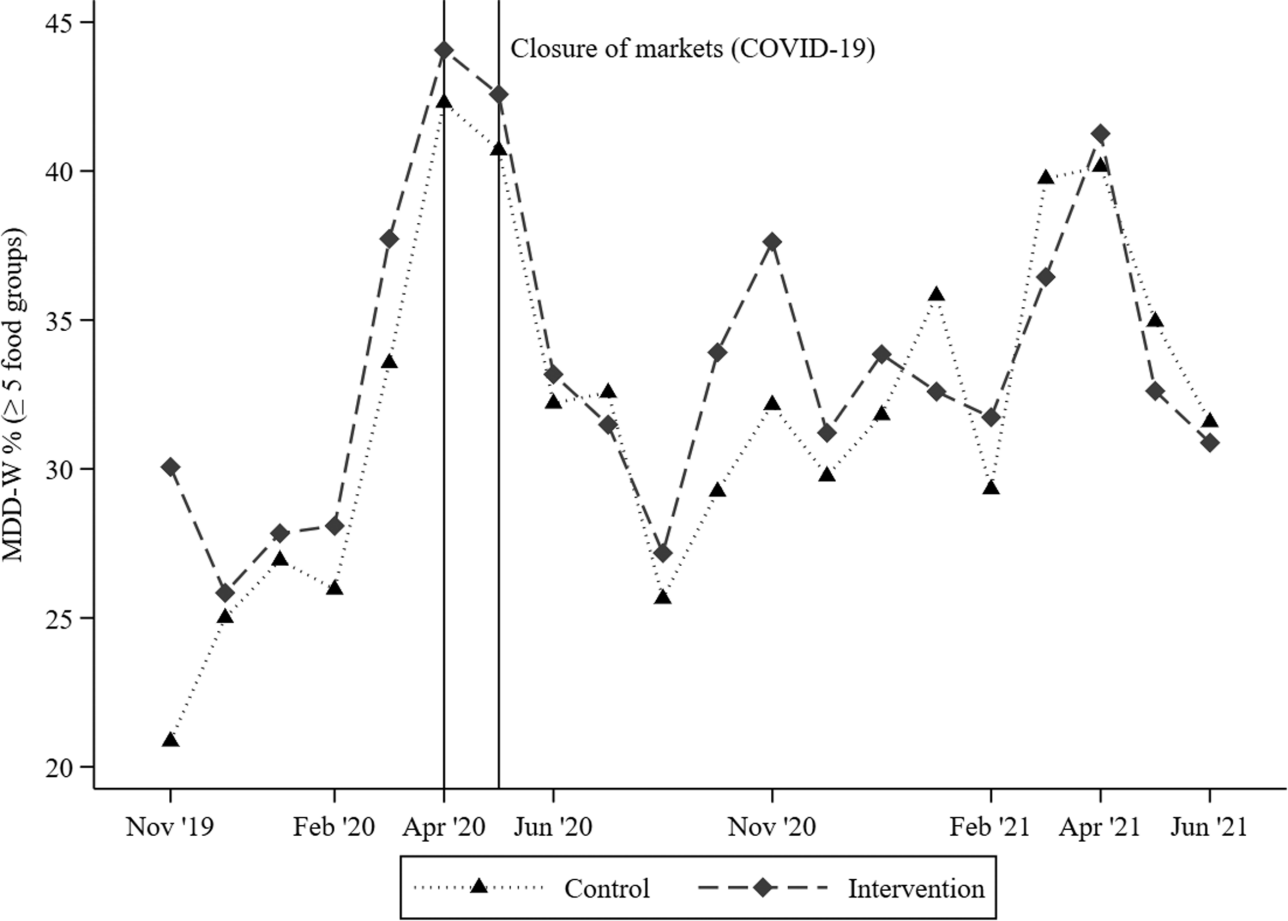
Proportion of pregnant women (*n* = 1757) achieving MDD-W, by study month and trial arm. The y-axis values range between a minimum of 20% and maximum of 45%. October 2019 (*n* = 8 women), July 2021 (*n* = 14), and August 2021 (*n* = 1) were not plotted due to the limited number of data points. All 2-sided Welch's independent-sample *t*-tests were nonsignificant by intervention arm (*P* > 0.05). Abbreviation: MDD-W, Minimum Dietary Diversity for Women.

Over the prenatal follow-up, in study months with a sample size ≥15 women, the mean FG score varied from 3.5 in December 2019 and August 2020 to 4.3 in April 2021 (**Table 2**; **Figure 1**). Similarly, the proportion of women achieving MDD-W varied between 28.2% in August 2020 and 43.4% in April 2020 (Table 2; **Figure 2**), whereas the prevalence of women consuming at least 6 FGs was always <25% (Table 2). No significant differences in the average monthly food group diversity score or MDD-W prevalence were observed between the intervention and control groups (all *P*_Welch_ values > 0.05). The monthly variability in the FG diversity score was explained more by the between-woman SD (*n* = 1757) than the within-woman SD [*n* = 1743; i.e., 1.3 compared with 0.7 FGs, respectively; intra-cluster correlation coefficient (*ρ*) = 0.73; footnote of Table 2]. Similarly, the between-woman SD of achieving MDD-W was 31.9 pp, whereas the within-woman SD was 23.1 pp, over an average of 6.2 months of follow-up (*ρ* = 0.68). Regarding individual FG consumption, starchy staples (>95%) and dark green, leafy vegetables (∼80%) were consumed by almost all pregnant women during each study month, whereas eggs and dairy products were consumed by <6% and <17% of pregnant women, respectively (Table 2; **Supplemental Figure 2**). Furthermore, over the prenatal follow-up, nuts and seeds and flesh foods were consumed on a regular basis (37% and 54%, respectively), whereas vitamin A–rich fruit and vegetable consumption was highly seasonal, with peaks between March and June (>25%; Table 2; Supplemental Figure 2). In addition, using nonaggregated, qualitative FG intake data (70,972 daily observations; mean ± SD, 40.4 ± 20.7 recalls per woman), we estimated that 5 recalls allows the observed FG diversity score to lie within 15% (±∼0.5 FG) of the true mean in 90% of the estimations (i.e., mean FG intake, 3.9; within-subject SD, 0.8). Our findings were stable among women with recall data from dry-season (5838 daily observations; *n* = 236) or rainy-season (663 daily observations; *n* = 53) months only and from those with recall data across both seasons (64,472 daily observations; *n* = 1468).

**TABLE 2:** FG diversity score and the proportion of women consuming individual FGs and achieving MDD-W during pregnancy, by study month^1^

	Oct ‘19	Nov ‘19	Dec ‘19	Jan ‘20	Feb ‘20	Mar ‘20	Apr ‘20	May ‘20	Jun ‘20	Jul ‘20	Aug ‘20	Sept ‘20	Oct ‘20	Nov ‘20	Dec ‘20	Jan ‘21	Feb ‘21	Mar ‘21	Apr ‘21	May ‘21	Jun ‘21	Jul ‘21	Aug ‘21^2^	Total
All starchy staples	100	95.8	96.4	97.5	98.4	97.9	96.6	95.9	95.7	97.6	97.3	97.6	98.2	98.4	97.9	97.3	97.3	97.7	98.9	98.9	98.1	100	100	97.3
Beans and peas	75.0	31.2	21.3	18.0	15.7	13.8	10.9	11.5	11.9	14.9	13.6	15.5	20.6	25.1	21.1	17.7	17.2	15.7	14.1	11.8	9.41	11.9	0	16.5
Nuts and seeds	50.0	43.5	39.7	37.8	33.4	36.0	34.0	31.8	30.5	32.3	30.9	41.5	48.9	48.5	42.8	40.9	38.3	31.6	37.0	31.9	38.4	26.3	0	37.4
Dairy	25.0	11.3	10.8	13.2	10.6	11.9	12.2	10.0	9.92	12.6	13.2	13.3	12.3	12.7	13.9	13.9	14.0	16.9	15.1	15.3	12.3	14.3	0	12.4
Flesh foods	62.5	49.8	54.6	54.1	53.5	56.1	57.9	59.8	51.8	54.0	55.6	51.7	51.6	52.4	54.3	57.9	54.2	58.0	54.0	58.4	58.7	54.8	100	54.2
Egg	12.5	5.13	3.47	3.75	2.34	3.42	2.99	3.81	3.97	4.42	4.61	3.99	3.96	3.68	4.18	3.89	2.91	4.03	2.99	3.70	4.31	4.17	0	3.84
Dark green, leafy vegetables	75.0	74.7	77.7	78.6	82.9	84.1	84.2	83.6	86.4	91.3	90.3	89.0	87.4	87.3	86.1	86.7	86.0	84.3	86.3	87.9	90.8	86.7	100	85.8
Vitamin A–rich fruits and vegetables	12.5	7.09	7.77	10.1	10.5	25.4	48.5	47.4	32.8	17.8	7.19	6.14	7.59	8.55	10.5	11.8	12.1	29.0	44.3	33.6	26.7	8.41	0	20.0
Other vegetables	62.5	44.3	45.1	56.8	61.9	64.2	66.0	55.5	35.8	30.4	42.1	62.6	63.7	48.6	51.2	63.5	67.7	68.4	65.9	57.4	49.6	44.3	0	53.9
Other fruits	12.5	17.3	19.7	15.2	14.8	13.8	11.1	12.5	24.2	32.6	22.0	15.1	15.7	16.9	19.7	18.8	17.0	15.1	13.6	12.9	13.9	33.5	0	17.8
FG diversity score (0–10)^3^	4.0 (4.0, 5.0)4.9 ± 2.2	3.7 (2.7, 4.7)3.8 ± 1.7	3.5 (2.8, 4.5)3.8 ± 1.4	3.7 (2.8, 4.6)3.8 ± 1.4	3.7 (3.0, 4.5)3.8 ± 1.3	4.0 (3.0, 5.0)4.1 ± 1.4	4.2 (3.0, 5.1)4.2 ± 1.5	4.0 (3.0, 5.0)4.1 ± 1.3	3.7 (2.8, 4.6)3.8 ± 1.4	3.6 (2.8, 5.0)3.9 ± 1.5	3.5 (2.8, 4.6)3.8 ± 1.4	3.8 (3.0, 4.8)4.0 ± 1.4	4.0 (3.1, 4.9)4.1 ± 1.4	3.9 (3.0, 5.0)4.0 ± 1.5	3.8 (3.0, 5.0)4.0 ± 1.6	4.0 (3.0, 5.0)4.1 ± 1.5	4.0 (3.0, 4.8)4.1 ± 1.5	4.0 (3.0, 5.0)4.2 ± 1.5	4.3 (3.5, 5.0)4.3 ± 1.4	3.9 (3.0, 5.0)4.1 ± 1.6	4.0 (3.0, 4.6)4.0 ± 1.4	3.4 (3.0, 4.8)3.8 ± 1.5	3.0 (3.0, 3.0)3.0	3.9 (3.1, 4.7)4.0 ± 1.3^5^
MDD-W	37.5	29.8	29.3	31.0	30.6	38.1	43.4	40.1	31.7	33.3	28.2	32.6	36.3	32.9	34.2	36.1	32.8	39.3	42.0	35.6	32.2	26.9	0	34.4^6^
≥6 FGs	12.5	13.9	12.9	15.2	13.2	19.6	23.0	20.1	16.3	16.7	13.3	14.7	16.9	16.2	18.2	18.8	16.8	20.1	21.1	19.1	13.6	12.1	0	17.0
Sample size^4^	8 (50.0)	112 (50.9)	198 (50.5)	299 (50.8)	439 (48.5)	576 (50.0)	587 (48.2)	810 (50.5)	816 (49.6)	740 (48.5)	783 (49.3)	875 (49.3)	906 (49.4)	829 (49.5)	740 (48.9)	669 (48.4)	598 (48.0)	266 (49.6)	367 (47.1)	224 (47.8)	98 (44.9)	14 (50.0)	1 (100)	1,757 (50.8)

1 Data are percentages unless otherwise stated. Abbreviations: FG, food group; MDD-W, Minimum Dietary Diversity for Women.

2 The last prenatal, qualitative, list-based, 24-hour FG recall was on August 6, 2021.

3 Median (*P*^25^, *P*^75^) and mean ± SD.

4 Number (% intervention arm).

5 The monthly aggregated between-person SD was 1.3 (*n* = 1757), the within-person SD was 0.7 food groups (*n* = 1743), and the mean number of monthly observations was 6.2.

6 The monthly aggregated between-person SD was 31.9 percentage points (*n* = 1757), while the within-person SD was 23.1 percentage points (*n* = 1743).

**FIGURE 3:**
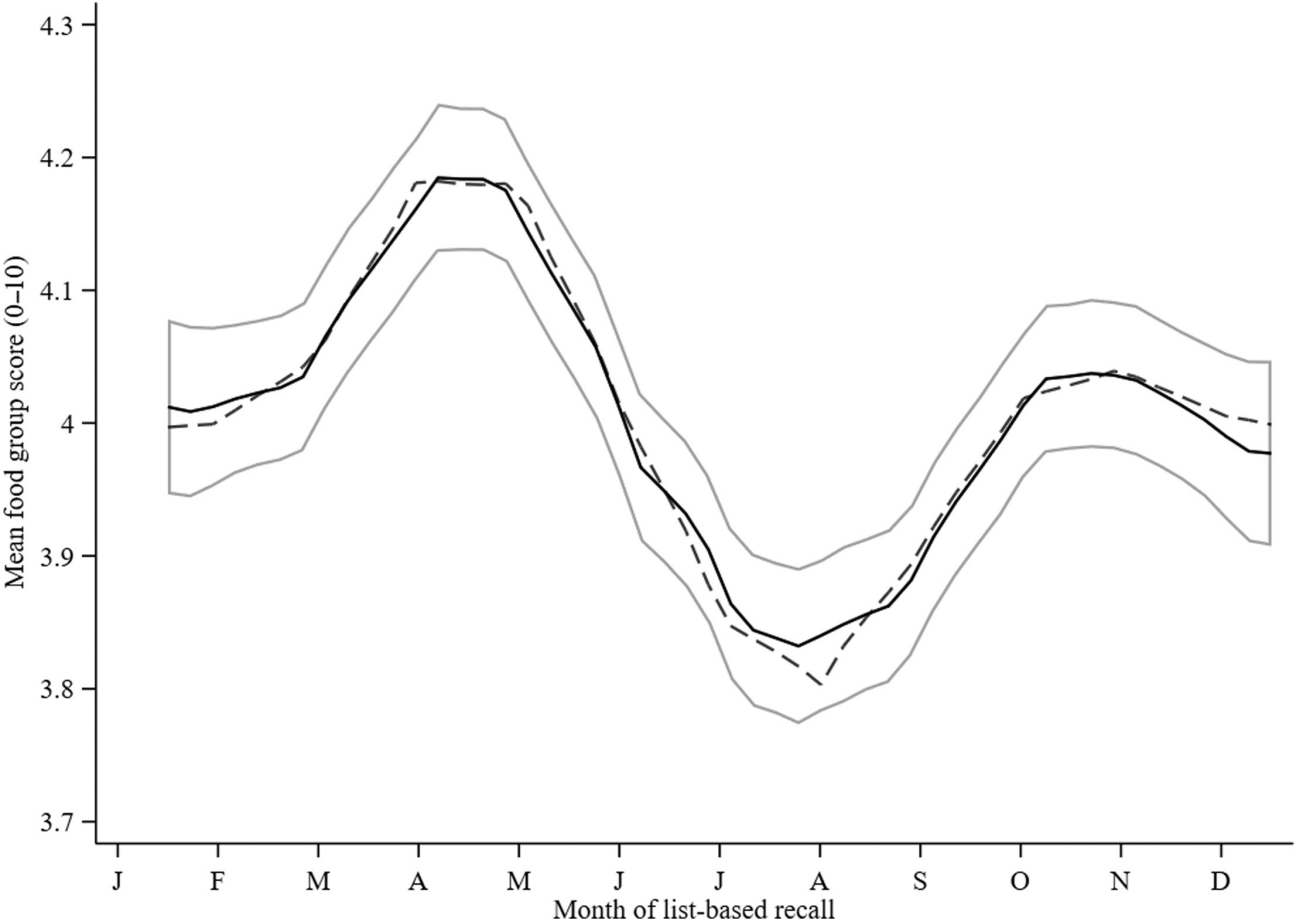
Monthly means and seasonal variation in a 10-point food group diversity score (*n* = 1757 women; *n* = 10,955 data points) in the MISAME-III trial. The y-axis ranges between a minimum of 3.7 and maximum of 4.3 food groups. The solid lines represent the local polynomial smoothing prediction of the monthly mean with 95% CI, whereas the dashed line represents the modeled seasonal variation, with Fourier series. The food group diversity score was fitted to the first-, second-, and third-order Fourier pairs. Abbreviation: MISAME-III, Micronutriments pour la Santé de la Mère et de l'Enfant study 3.

**FIGURE 4:**
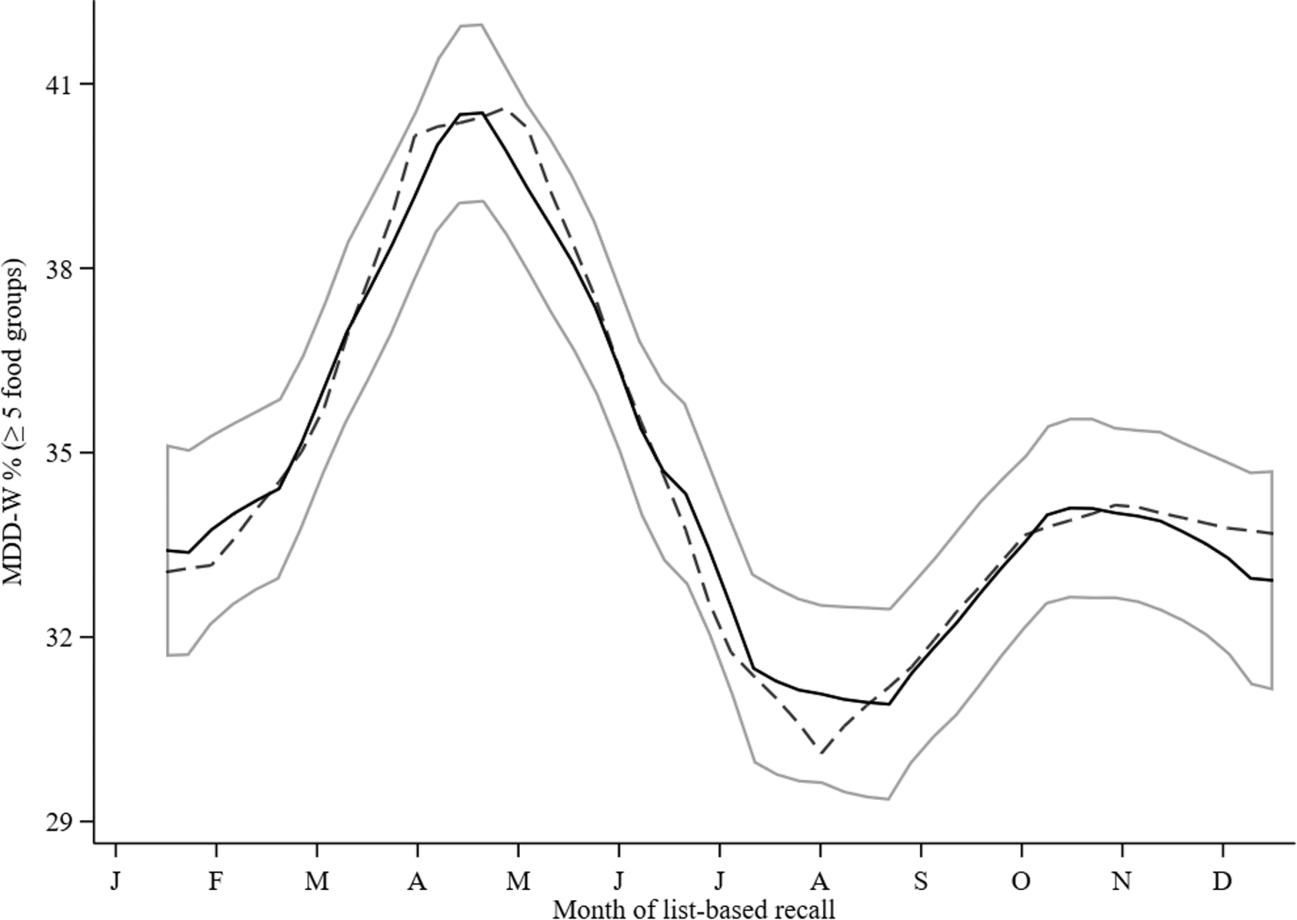
Monthly proportion of pregnant women achieving MDD-W (*n* = 1757 women; *n* = 10,955 data points) in the MISAME-III trial. The y-axis ranges between a minimum of 29% and maximum of 42%. The solid lines represent the local polynomial smoothing prediction of the monthly mean proportion with 95% CI, whereas the dashed line represents the modeled seasonal variation, with Fourier series. The MDD-W was fitted to the first-, second-, and third-order Fourier pairs. Abbreviations: MDD-W, Minimum Dietary Diversity for Women; MISAME-III, Micronutriments pour la Santé de la Mère et de l'Enfant study 3.

The comparison of the goodness of fit of different seasonality models with increasing order is presented in **Table 3**. The models provide evidence (i.e., *P*_LR χ²_ <0.05 and lowest AIC and BIC) that the first 3 pairs of Fourier terms (i.e., F_3_ model) are necessary to explain the seasonality in the FG diversity score and MDD-W. Both the FG diversity score (*P*_LR χ²_ <0.0001) and the MDD-W (*P*_LR χ²_ <0.0001) were significantly associated with the month of the list-based FG recall. From the fitted Fourier models, it can be concluded that dietary diversity showed modest monthly variations. The FG diversity score and MDD-W reached their zeniths at the end of the dry season—more precisely, in April (4.3 ± 1.5 FGs and 42.8% ± 40.3%, respectively)—whereas their nadirs both appeared in the rainy season in August (3.8 ± 1.5 FGs and 28.2% ± 37.7%, respectively; **Figures 3** and **4**).

**TABLE 3:** Comparison of the goodness of fit of seasonality models for a 10-point FG diversity score and the MDD-W indicator in the MISAME-III trial^1^

	FG diversity score		MDD-W	
Model comparison^2^	LR χ² (df)	*P*	LR χ² (df)	*P*
F_0_–F_1_	112 (2)	<0.0001	109 (2)	<0.0001
F_1_–F_2_	167 (2)	<0.0001	112	<0.0001
F_2_–F_3_^3^	7.01	0.03	9.20	0.01

1 Values are the likelihood ratio chi-squared test statistic and its corresponding df of model comparisons for the FG diversity score and MDD-W (*n* = 1757 women; *n* = 10,936 monthly observations). The dependent variables in the models are the FG diversity score and MDD-W; the independent variables are Fourier terms. Abbreviations: FG, food group; LR χ², likelihood ratio chi-squared test statistic; MDD-W, Minimum Dietary Diversity for Women; MISAME-III, Micronutriments pour la Santé de la Mère et de l'Enfant study 3.

2 F_0_ is the intercept-only model. F_1_ is a model including only the first-order Fourier pair, whereas F_2_ also includes the second-order pair and F_3_ additionally includes the third-order pair.

3 F_3_ has the lowest Akaike and Bayesian information criteria.

## Discussion

We report that dietary diversity, as measured by a 10-point FG diversity score and the MDD-W prevalence, was consistently low (mean monthly values, <5 FGs and <45%, respectively). Furthermore, an estimated 5 qualitative, list-based recalls were required to reasonably estimate a woman's usual FG diversity score. A Fourier-transformed regression analysis showed that women's dietary diversity reached a zenith at the end of the dry season, whereas the nadir was in the rainy season in Burkina Faso. To our knowledge, this is the first study to demonstrate seasonal patterns in the FG diversity score using high-frequency, longitudinal data from pregnant women collected across multiple years.

Though we concluded that intracluster correlation coefficients of the prenatal FG diversity score and MDD-W prevalence were high (i.e., both *ρ* values ∼0.70; thus, within-woman changes in dietary diversity were low), defining the (absolute) magnitude of meaningful changes in dietary diversity remains speculative. To illustrate, Martin-Prével et al. (57) reported that an urban household's average dietary diversity score significantly decreased by ∼0.5 FGs following the 2008 food price crisis. Furthermore, Hanley-Cook et al. (58) arbitrarily set noninferiority of the MDD-W prevalence, estimated by proxy methods as compared to weighed food records, at ≤5 pp. Over the MISAME-III efficacy trial's follow-up, the seasonal differences in the FG diversity score and MDD-W prevalence were in the order of 0.5 FG and 2.6 pp, respectively, between the peak and trough. However, evidence is lacking as to whether these differences should be considered relevant for women's prenatal nutritional status.

Unsurprisingly, the consumption of cereals, roots, and plantains was high, and the FG diversity score and MDD-W prevalence were low across all seasons. These findings have been previously observed in both rural (10,19, 49) and urban samples of Burkinabe women (59, 60). Indeed, low dietary diversity has been attributed to a high dependence on *tô*, which is a stiff, cereal-based porridge served with a watery sauce containing green, leafy vegetables (e.g., sorrel and baobab leaves) and sporadically garnished with other vegetables (e.g., eggplant, tomato, and okra) or nuts and seeds (peanuts and néré seed), with or without meat, fish, or caterpillars (19,27,43). Moreover, in parallel to our findings, vitamin A–rich fruit and vegetable consumption [e.g., ripe mangoes (2)] exhibited the most seasonality among under-5 children in Nepal, Peru, and Senegal (8), which was, however, buffered slightly by a higher consumption prevalence of flesh foods and other vegetables by women enrolled in MISAME-III. In contrast, the lack of intra-annual variability in the consumption of dark green, leafy vegetables, for example, might be explained by the different seasonal harvesting patterns of species and varieties within the same FG (61) or by processing techniques (e.g., dried compared with fresh foods). Our study also indicated that the MDD-W prevalence was relatively stable between August and January, and then increased from February through May. Similar findings by Lourme-Ruiz et al. (19) attributed the rise in the FG diversity score to increased purchases of onions, tomatoes, and mangoes, and identified peaks in March to June as periods of high food purchases and concomitant foraging of fruits (e.g., shea nuts, plums, and wild grapes).

Our panel data, which covered the entire 23 months of prenatal follow-up, corroborate key results from a recent, cross-sectional substudy of the MISAME-III efficacy trial. In brief, de Kok et al. (27) conducted single-day, quantitative, 24-hour recalls among a random sample of 470 pregnant women and also reported low dietary diversity (and nutrient intakes) between September and October 2020, in part due to low egg, dairy, flesh food, and fruit consumption. Moreover, in the preharvest season, fortified BEP and IFA supplements did not displace nutrient intakes among pregnant women when compared to IFA alone (27). Likewise, the present study's findings indicate that the average monthly FG diversity scores were not significantly different between the intervention and control groups over the prenatal follow-up.

Lastly, our longitudinal study revealed that seasonal patterns (e.g., dry and rainy seasons) modestly affect maternal dietary diversity at the population level. Hence, mismatches in the timing of repeated cross-sectional surveys might exert spurious influences on inferences about changes in the FG diversity score or MDD-W prevalence among pregnant women over time, in particular if observational studies are not conducted in the same season. Thorne-Lyman et al. (8) indicated that average annual rates of change (over ∼5 years) in children's 8-point FG score and a dichotomous indicator (≥5 FGs) were not markedly larger than the average seasonal changes observed, which suggests that seasonal fluctuations are large enough to introduce bias into inferences of change and trends in dietary diversity, particularly when data collection is annual and baseline levels are high. Furthermore, in MISAME-III, a dichotomous MDD-W (i.e., only crossing >5 FGs reflects change) resulted in only a slight loss of responsiveness to seasonality among MISAME-III participants, thus leading us to conclude that changes occur mainly amongst segments of our sample with FG diversity scores close to the 5-FG cutoff.

Our study has some limitations that warrant caution. First, dietary diversity in isolation is not sufficient to reflect diet quality, which requires adequate and balanced macronutrient intakes and moderation in intakes of free sugars, sodium, and certain lipids (62). Second, qualitative, list-based, 24-hour recalls have been shown to overestimate (i.e., in particular, via false positives) the consumption of specific FGs, the FG diversity score, and the proportions of women achieving MDD-W, as compared to a weighed food record, among women of reproductive age in Cambodia, Ethiopia, and Zambia (58). Nonetheless, Nguyen et al. (63) found no differences in performance in predicting MPAs of the FG diversity scores or MDD-W measured between list-based and quantitative, open, 24-hour recalls among pregnant women in Bangladesh and India. Furthermore, the 10-point FG diversity score was significantly associated with the MPA among pregnant women and adolescents, although a 6-FG cutoff was required to improve the classification of women with an MPA >0.60 (63). In contrast, the proportion of pregnant women reaching an MPA >0.60 was too low to assess any FG diversity score cutoff points in Burkina Faso (55). Third, the aggregation of FG consumption data by study month, due to an aforementioned software error, slightly reduced the variability of our within-woman dietary diversity estimates. Fourth, seasonality might have been underestimated, simply because our dietary diversity indices captured consumption between each FG, but are inherently unresponsive to changes in actual quantities consumed or within-FG diversity or richness over the previous day and night.

In conclusion, we provide evidence that *1*) 5 list-based, 24-hour recalls are sufficient to estimate a pregnant woman's usual qualitative FG diversity score; and *2*) seasonal patterns modestly affect the average monthly FG diversity score and the proportion of women reaching MDD-W in Houndé, Burkina Faso. These findings imply that repeated, cross-sectional food consumption surveys must take into account timing to ensure nonbiased inferences of change and trends at a group level.

## Supplementary Material

nxac104_Supplemental_File

## Data Availability

Researchers who provide a scientifically sound proposal will be allowed access to the deidentified individual participant data. To gain access, data requesters will need to sign a data access agreement. These proposals will be reviewed by the principal investigator. Supporting study documents, including the protocol and questionnaires, are publicly available on the trial's website: https://misame3.ugent.be/.

## Notes

This work was supported by the Bill & Melinda Gates Foundation (grant number OPP1175213).

Author disclosures: The authors report no conflicts of interest.

The funder had no role in study design, data collection and analysis, decision to publish, or preparation of the manuscript.

Supplemental Table 1 and Supplemental Figures 1 and 2 are available at the “Supplementary data” link in the online posting of the article and from the same link in the online table of contents at https://academic.oup.com/jn/.

Abbreviations used: AIC, Akaike information criterion; BEP, balanced energy-protein; BIC, Bayesian information criterion; FG, food group; IFA, iron-folic acid; LMIC, low- and middle-income country; MDD-W, Minimum Dietary Diversity for Women; MISAME-III, Micronutriments pour la Santé de la Mère et de l'Enfant study 3; MPA, mean probability of adequacy; pp, percentage point.
