# Correction

**DOI:** 10.1093/jn/nxac193

**Published:** 2022-09-06

Correction to Hanley-Cook et al. Seasonality and Day-to-Day Variability of Dietary Diversity: Longitudinal Study of Pregnant Women Enrolled in a Randomized Controlled Efficacy Trial in Rural Burkina Faso. J Nutr 2022;152(9):2145–54.

This is a correction to: Giles T Hanley-Cook, Alemayehu Argaw, Brenda de Kok, Laeticia Celine Toe, Trenton Dailey-Chwalibóg, Moctar Ouédraogo, Patrick Kolsteren, Lieven Huybregts, Carl Lachat, Seasonality and Day-to-Day Variability of Dietary Diversity: Longitudinal Study of Pregnant Women Enrolled in a Randomized Controlled Efficacy Trial in Rural Burkina Faso, *The Journal of Nutrition*, 2022;152(9):2145–54.

In the originally published version of this manuscript, figure 1 was inadvertently duplicated in figure 2. The correct figure 2 is now available in the article and below.

**Figure:**
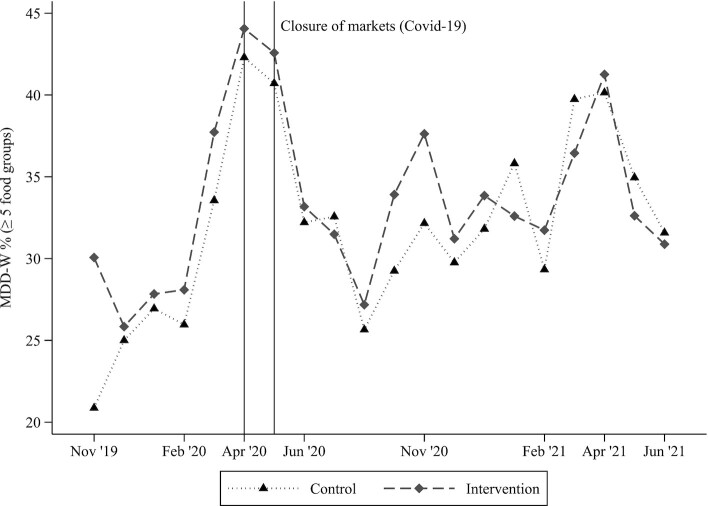


The publisher apologizes for this error.

## Notes

Address correspondence to GTH-C (e-mail: gilestcook@gmail.com).

