# Supplementary material for: Seasonality and Day-to-Day Variability of Dietary Diversity: Longitudinal Study of Pregnant Women Enrolled in a Randomized Controlled Efficacy Trial in Rural Burkina Faso
Source: J Nutr. 2022 May 7;152(9):2145–54. doi: 10.1093/jn/nxac104 (PMC9445846; doi:10.1093/jn/nxac104)
Supplement: nxac104_Supplemental_File [file nxac104_supplemental_file.pdf]

Supplemental Table 1. Food list for list-based recall method in Houndé, Burkina Faso[1]

**Food group**		**Corresponding food and drinks**
**i.**	**Cereals, roots, tubers, and plantains**	White sorghum, red sorghum, millet, small millet, rice, corn, pasta (macaroni, spaghetti, …), wheat (bulgur, bread, ...), fonio, white sweet potato, potato, yam, taro, other white tubers (fabirama), manioc (atiéké, gari), plantain (alloco) (**except sweet potato with orange flesh**)
**ii.**	**Legumes**	Beans (cowpeas), ground peas, peas, chickpeas, lentils, zamné, other legumes
**iii.**	**Nuts and seeds**	Peanuts (in paste or other), sesame, cashew nuts, wild nuts, cottonseeds, palm seeds (not red), ...
**iv.**	**Dark green leafy vegetables**	Sorrel, amaranth leaves, baobab leaves, bulvaka, spinach, onion leaves, bean leaves, cassava leaves, potato leaves, wild green leaves, …
**v.**	**Vitamin A rich vegetables**	Carrot, red pepper, orange squash, sweet potato with orange flesh
**vi.**	**Other vegetables**	Cabbage, okra, green pepper, fresh tomato (except concentrate), eggplant, onions, green beans, avocado, kapok, zucchini, cucumbers, turnips, …
**vii.**	**Vitamin A rich fruits**	Mango, papaya, néré (fruit or flour), fresh juice of these fruits
**viii.**	**Other fruits**	Banana, orange, lemon, pineapple, monkey bread, guava, jujube, melon, sugar cane, apple-cinnamon, wild fruits (grapes, tamarind, shea butter, ...), fresh juice of these fruits
**ix.**	**Eggs**	Chicken, guinea fowl, and quail eggs
**x.**	**Meat, poultry, offal, insects, and fish**	Beef, mutton, goat, pork, tongue, rabbit, bush meat, chicken, guinea fowl, offal (tripe, liver, kidney, oxtail kidney, oxtail, ...), insects (caterpillars, ...), fresh; smoked; and salted fish, canned tuna, all kinds of seafood (**except a pinch of fish powder**)
**xi.**	**Dairy products**	Fresh milk, powdered milk, concentrated milk (sweetened or not), yogurt, cheese, fresh cream

[1]See original version on p.7: https://misame3.ugent.be/resource-files/MISAME-III_CSPRo_v3_may2020.pdf

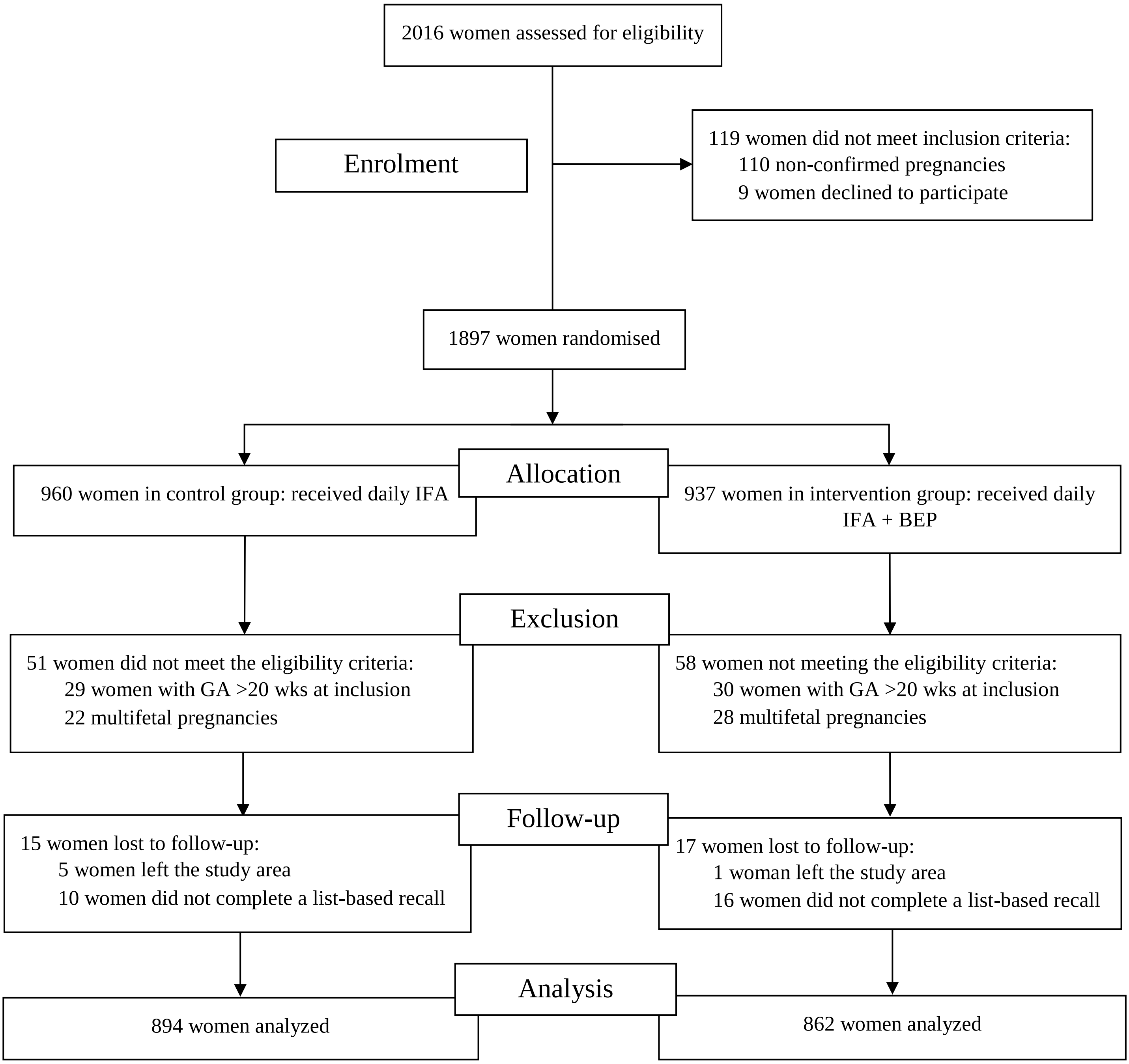

Supplemental Figure 1. Longitudinal study flowchart, by prenatal MISAME-III trial arm. BEP, balance energy-protein; GA, gestational age; IFA, iron-folic acid.

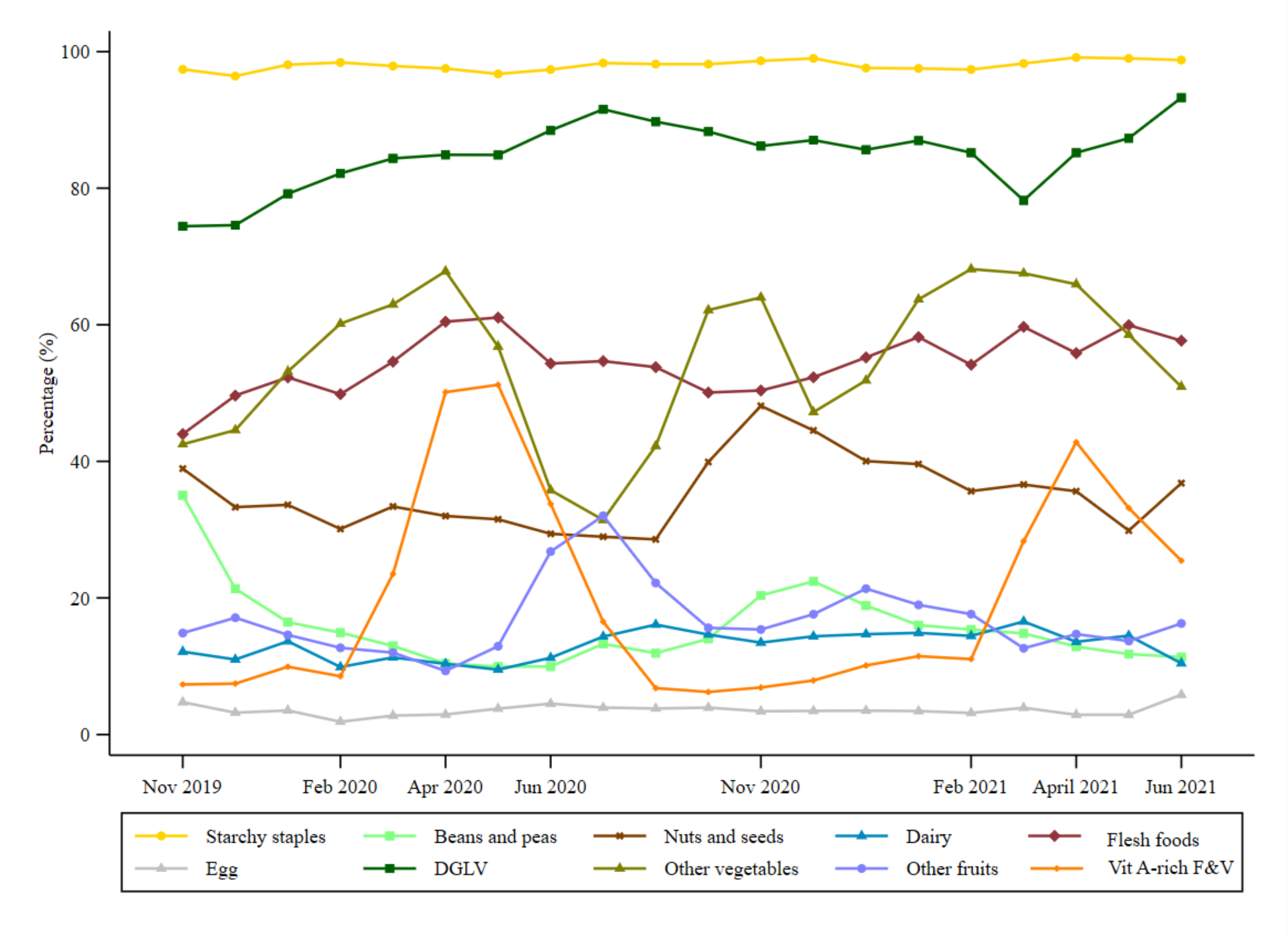

Supplemental Figure 2. Proportion of pregnant women consuming food groups, by study month. October 2019 (n = 8 women), July 2021 (n = 14), and August 2021 (n = 1) were not plotted due to the limited number of monthly data points. DGLV, dark green leafy vegetables; F&V, fruits and vegetables.
